# A proposed scenario to improve the Ncut algorithm in segmentation

**DOI:** 10.3389/fdata.2023.1134946

**Published:** 2023-03-03

**Authors:** Nhu Y. Tran, Huynh Trung Hieu, Pham The Bao

**Affiliations:** ^1^Faculty of Information Technology, Industrial University of Ho Chi Minh City, Ho Chi Minh City, Vietnam; ^2^Information Technology Faculty, Ho Chi Minh City University of Food Industry, Ho Chi Minh City, Vietnam; ^3^Information Science Faculty, Sai Gon University, Ho Chi Minh City, Vietnam

**Keywords:** GPU, CPU, parallel computing, Ncut, FCM

## Abstract

In image segmentation, there are many methods to accomplish the result of segmenting an image into k clusters. However, the number of clusters k is always defined before running the process. It is defined by some observation or knowledge based on the application. In this paper, we propose a new scenario in order to define the value k clusters automatically using histogram information. This scenario is applied to Ncut algorithm and speeds up the running time by using CUDA language to parallel computing in GPU. The Ncut is improved in four steps: determination of number of clusters in segmentation, computing the similarity matrix W, computing the similarity matrix's eigenvalues, and grouping on the Fuzzy C-Means (FCM) clustering algorithm. Some experimental results are shown to prove that our scenario is 20 times faster than the Ncut algorithm while keeping the same accuracy.

## 1. Introduction

Image segmentation is a key step for grouping objects which have the same characteristics or properties such as color, intensity, or texture. In an image, each object is called the domain—Region and its outlines are called the boundary. Feature vectors of regions are created based on their properties and are used to distinguish them. Image segmentation describes details of the various components in image to classify and recognize objects easier (Nock and Nielsen, 2004; Starck et al., 2005; Yu et al., 2009; Belahcene et al., 2014; Dhanachandra et al., 2015; Minaee and Wang, 2019). For instance, based on image segmentation, face detection contributes to better face recognition and user identification.

In the segmentation process of image with high-resolution data, the parallel computation on the image data is divided into two approaches. Approach to parallel programming model with hybrid model on CPU - GPU (Agulleiro et al., 2012) is a powerful co-processor system because the CPU and GPU have the combined properties of using both types of additional processors allowing for the execution of many large applications for optimal performance. Specifically, OpenMP, CUDA, and MPI libraries are used on CPUs and GPUs (Sirotković et al., 2012; Baker and Balhaf, 2017; Fakhi et al., 2017; Dalvand et al., 2020; Wang N. et al., 2020). Clustering in a multi-core architecture starts with dividing the image data into regions in a grid pattern, and then parallelizes the segmentation over the regions. Parallel file system approach with Hadoop, MarReduce, and Spark (Augustine and Raj, 2016; Li et al., 2016; Cao et al., 2018; Liu et al., 2019; Wang X. et al., 2020) is built for high resolution image segmentation. All of the above approaches allow increasing the performance of the algorithm.

Among the several segmentation algorithms, Ncut algorithm (Shi and Malik, 2000) is one of the efficient algorithms for image segmentation, which is based on graph theory. It detects the boundary between two regions by partitioning and grouping based on not only local features of image but also global features of image. In this algorithm, a distinct parameter for dividing the input image into different regions was calculated. However, in graph theory, dividing graph problem is an NP-complete problem meaning that it cannot be solved in polynomial time. Besides, the complexity of the Ncut algorithm is affected by the size of the input image. On that basis, for the purpose of improving the processing time of image segments, several parallelization methods have been applied. Shiloach and Vishkin (1982) developed the first parallel algorithm based on the breadth-first search algorithm. Anderson and Setubal (1992) use parallel in the numbering algorithm on workstations for better computation. Wassenberg et al. (2009) used the Minimum Spanning Tree with optimal function calculation and parallel execution on machines sharing memory. XianLou and ShuangYuan implemented Ncut algorithm in parallel on GPUs. Therefore, the performance of these methods depends on the size of each small area. The right number of divisions and the proper size is a problem (XianLou and ShuangYuan, 2013). However, these methods mostly implement parallel algorithms on each small partition of the image. That means the image is initially divided into many small regions and then applying the segmentation algorithm on small areas in parallel.

The execution time of Ncut algorithm is *O*(*MxN*) in which *N* is the number of pixels that is equivalent to the number of nodes of the graph created by image. Besides, M is the number of steps that Lanczos algorithm (Cullum and Willoughby, 2002) takes to find the eigenvalues in the process. Since every node of input image only relates with some neighbors, W matrix can be stored as a sparse matrix which is efficient usage memory. Moreover, because computing the similarity matrix W and its eigenvalues take too much time, we propose a parallel computing method using CUDA on GPU for solving Ncut problem.

Our paper is organized into three main sections, the first section is the introduction of our approach, which is discussed in this paper. The second section describes our proposed method in detail. This section consists of three subsections: determination of number of clusters in segmentation, computing the similarity matrix W, computing the similarity matrix's eigenvalues, and grouping on the FCM. The last section is about some experimental results in comparing the speed time between our approach and some conventional approaches and comparison of accuracy in segmentation.

## 2. Proposed method

Ncut method proposed by the group author Shi and Malik is as follow:

+ Given an image, set up a weighted graph *G* = (*V, E*), where the vertex set V of graph are the point in the feature space, every edge in the edge set E is formed between every pair of nodes, and set the weight on the edge connecting two nodes to be a measure of the similarity between the two nodes.
+ Solve (*D*−*W*)*x* = λ*Dx* for eigenvector with the smallest eigenvalues. Where, D is an *N*×*N* diagonal matrix, W is an *N*×*N* symmetrical matrix, *x* is an eigenvector, and λ is an eigenvalue.
+ Use the eigenvector with the second smallest eigenvalue to bipartition the graph by finding the splitting point such that *Ncut* is minimized.
+ Decide if the current partition should be subdivided and recursively repartition the segment parts if necessary.
+ Recursively repartition the segment part if necessary.

The Ncut algorithm should be improved in the image segmentation problem for computational performance. Firstly, automatic k cluster prediction method is needed to choose the number of k partitions in image segmentation applications, we propose to predict the number k clusters based on the characteristic histogram of the image. Secondly, in one step of the algorithm, the K-means grouping method is used to group on the eigenvector set found. Since the eigenvector set is a real data set, there will be errors during the clustering calculation due to the computer's structure and numerical representation. We propose to use FCM algorithm with the expectation that it can fuzzify the data so that errors can be accepted for better data clustering prediction. Furthermore, the process of finding the similarity matrix and eigenvalues of the sequential execution problem takes up a considerable amount of execution time in the whole algorithm. We propose to apply parallel computation on GPU for this calculation step with the expectation of better computing performance on large image data.

### 2.1. Determination of number of clusters in segmentation

In most of segmentation problems, the issue of deciding the number of objects in order for segmentation is crucial and indispensable. Generally, this number of groups will be intuitively inputted based on user estimation. The estimation comes from viewing an arbitrary image and giving a number k.

We propose an automatic approach to deciding the number k of clusters based on histogram. The gray-level histogram provides many extreme points (minimum and maximum points). The exploration of number of maximum points is the key of deciding the number of clusters in which the following formula is satisfied (1).

Let δ_*i*_ ∈ *R* and define *f*:*R*^2^×*R*^2^→*R*^2^×*R*^2^ by


(1) $$\begin{array}{l} f\left(P_{1},\;P_{2}\right)=\{\left(P_{1},\;P_{2}\right)\;|\;\left(P_{1}\left(x\right)-P_{2}\left(x\right)\right)>\delta_{1}\;(P_{1}\left(y\right)\\ \;-P_{2}\left(y\right))>\delta_{2}\} \end{array}$$

Where (*P*_1_(*x*)−*P*_2_(*x*))>δ_1_ is the height deviation or the deviation of the total pixels in a gray-level between two peaks. And the (*P*_1_(*y*)−*P*_2_(*y*))>δ_2_ is the distance between two extreme points. The value of δ_1_ and δ_2_ is estimated by statistics from a pre-selected set of images.

**Figure 1:**
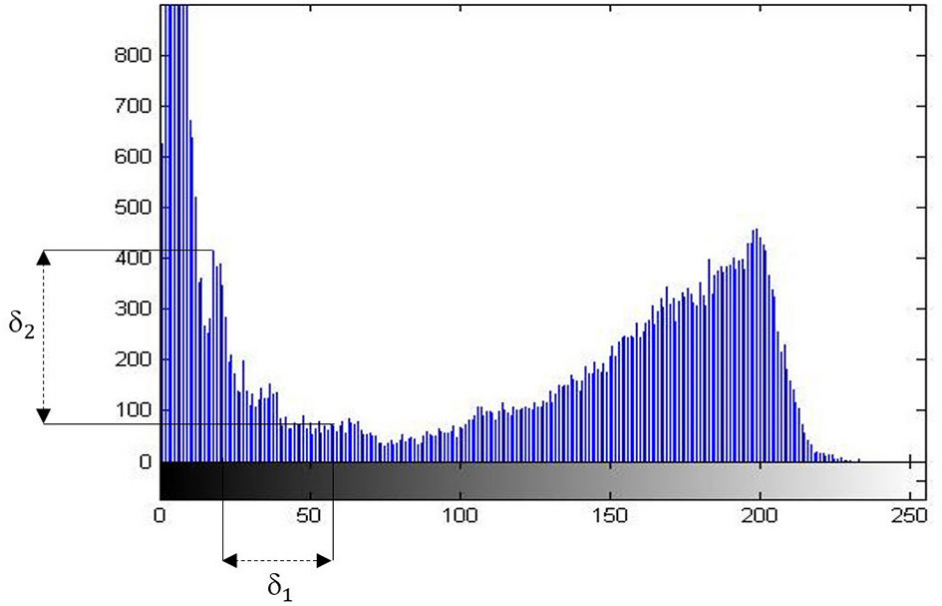
The histogram of image depicts the distance and differences.

The Figure 1 of gray-level histogram shows us the number of clusters based on distance and deviation.

Algorithm 1 is present to determine the number of segments in an image.

**Algorithm 1:** K-segment_Histogram.

**Input:** Array is gray-level histogram, δ_1_, δ_2_
**Output:** K image segments
K:=0
Step 1: Find the largest of local element P_1_
Step 2: Find the smallest of local element P_2_ such
that *P*_2_(*y*) < *P*_1_(*y*) and satisfy formula (1)
Step 3: K increases by 1 unit
Step 4: Repeat step 1

### 2.2. Computing the similarity matrix W

**Figure 2:**
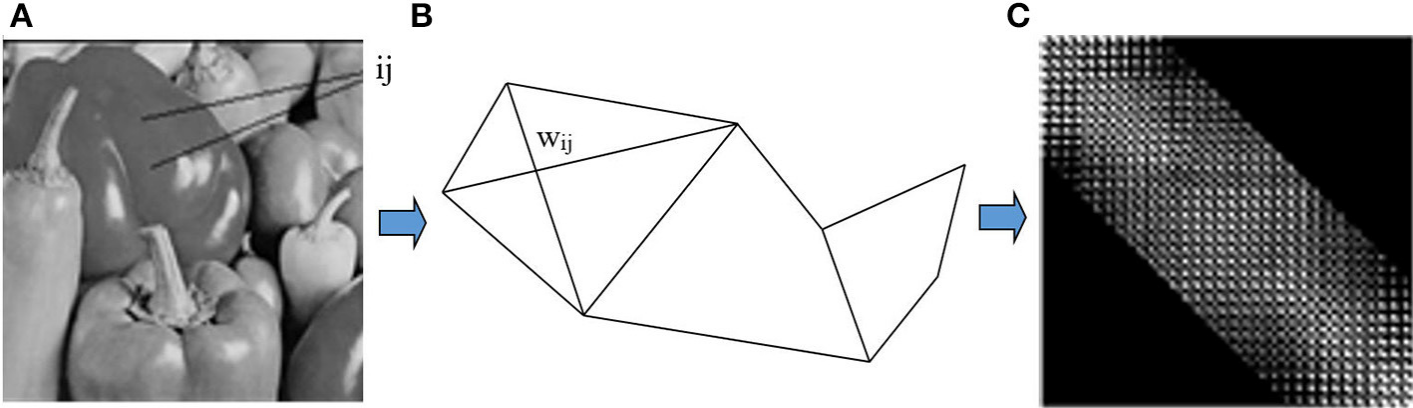
The graph and the similarity of original image: **(A)** Original image. **(B)** Grap G. **(C)** Matrix affinity W.

The similarity matrix W or the matrix affinity is the matrix representation of the relationship between the nodes in the original image. For example, the original image is converted to graph and W matrix as shown in Figure 2.

We apply the Malik and Shi grouping algorithm to image segmentation based on brightness. We construct the graph *G* = (*V, E*) by taking each pixel as a node and define the edge weight *w*_*ij*_ between node *i* and *j* as the product of a feature similarity term and spatial proximity term using formula (2).


(2) $$w_{ij}=e^{-\frac{{\left\|I\left(i\right)-I\left(j\right)\right\|}_{2}^{2}}{\sigma_{I}}}\times \left\{ \begin{array}{l} e^{-\frac{{\left\|X\left(i\right)-X\left(j\right)\right\|}_{2}^{2}}{\sigma_{X}}\;if\;{\left\|X\left(i\right)-X\left(j\right)\right\|}_{2}<r}\\ \;0\;otherwise \end{array}\right.$$

Where *X*(*i*) is the spatial location of node *i* and *I*(*i*) is the intensity value of the brightness. We have the weight *w*_*ij*_ = 0 for any pair of nodes *i* and *j* that are more than *r* pixel apart.

**Figure 3:**
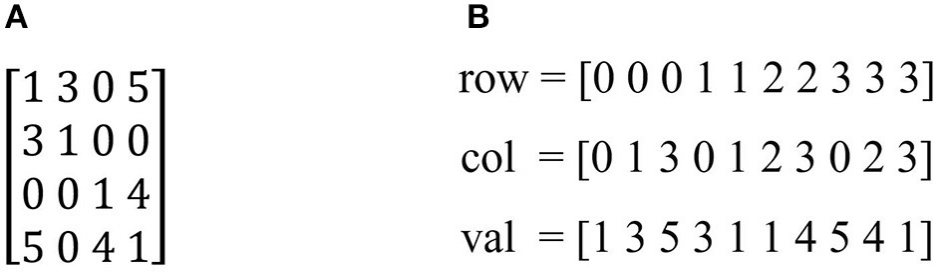
The COO form of input matrix: **(A)** Input matrix. **(B)** The COO format of input matrix.

As we see, *r* is often small than size of matrix image (Shi and Malik, 2000). Therefore, the numbers of zeros elements are more than other elements in the similarity matrix. In other words, matrix affinity W is a sparse matrix. To save memory usage, we stored W in form coordinate (COO) (Bell and Garland, 2008) which consist of element's indexes having nonzero elements. Specifically, the input matrix A contained into three arrays *row, col* and *val* corresponding with row index, column index and value of nonzero elements as shown in Figure 3.

For each pixel i, there is corresponding connected pixels j. In other words, there will be w_ij_ for two connected pixels i and j according to the given connection distance r. Thus, with the input image I(row × col), it will take a long time to consider the vertices sequentially (Shi and Malik, 2000). Therefore, we propose Algorithm 2 to parallelize each vertex iϵ(row × col) to find connected vertices j and calculate the weight w_ij_ using formula (2).

**Algorithm 2:** Sparse matrix_Ncut_GPU.

**Input:**Original image I; r is the radius; nrow,
ncol are sizes of the input image; dMaskx, dMasky,
dnMask are abscissa, ordinate and number of elements in the mask by Appendix A
**Output:**COO form of the W matrix (dVal, dRow, dCol)
Step 1: Copy image I, dMaskx, dMasky, and dnMask
from host (CPU memory) to device (GPU memory)
Step 2: Determine the total number of non-zero
elements of the weight matrix W corresponding to
the input matrix I by Algorithm 6
Step 3: Allocate the memory for variables
dRow, dCol, dVal are *n* × *sizeof*(*int*), *n*×*sizeof*(*int*),
*n*×*sizeof*(*double*)
Step 4: Build a grid of execution threads
(gridDim = (floor(((nrow × ncol)/r)^2^), 1, 1), blockDim = (r, r, 1)) Step 5: Call kernel function, execute parallelly
threads to calculate dVal, dCol, dRow:
Step 5.1: Determine the index of the threads
under execution
idx = blockidx.x^*^blockDim.x + threadIdx.x
Step 5.2: Calculate the pixel position under
consideration: $tx=\frac{idx}{ncol}$; *ty* = *idx%ncol*
Step 5.3: Calculate the neighboring points at
the pixel under consideration that will have
positions *x* = *tx*+*dMaskx*[*i*], *y* = *ty*+*dMasky*[*i*]*withi*∈
[0, .., *dnMask*] respectively
Step 5.4: Calculate the wij values by formula (2)
and store in the variables dVal, dRow, dCol

### 2.3. Computing the similarity matrix's eigenvalues

The Ncut problem to find eigenvalues of weight matrix W means finding k smallest eigenvectors of Laplace matrix based on weight matrix W. Since weight matrix W is a symmetric sparse matrix of relatively large size, it takes a lot of time to compute the eigenvector in the whole image segmentation process. Especially with the larger image size, the matrix size also increases exponentially, so it takes more time to find the eigenvalues in this case. We propose a method to parallelize the eigenvalues of the W matrix on the GPU and the Lanczos method (Cullum and Willoughby, 2002) as an effective algorithm for finding the k smallest eigenvalues. We compute Lanczos by paralleling in GPU. Each j, we compute parallel the multiplication between matrix and vector, the multiplication between vector and vector according to Algorithm 3.

**Algorithm 3:** Lanczos_Ncut_GPU.

**Input:**The symmetric matrix *A*_*n*×*n*_, k
**Output:**The k smallest eigenvalue and eigenvector
Step 1: Random vector $v_{1}\in R^{n}$ and ||*v*_1_||_2_ = 1
Step 2: *j* = 1, β_1_ = 0, *v*_0_ = 0
Step 3: We compute:
*w*_*j*_ = *Av*_*j*_ by Algorithm 8
α_*j*_ = *w*_*j*_*v*_*j*_ by Algorithm 7
*w*_*j*_ = *w*_*j*_−α_*j*_*v*_*j*_−β_*j*_*v*_*j*−1_ by Algorithm 9
β_*j*+1_ = ||*w*_*j*_ ||
*v*_*j*+1_ = *w*_*j*_/β_*j*+ 1_ *T*[*j, j*] = α_*j*_ *T*[*j*−1, *j*] = β_*j*+1_
*T*[*j, j*−1] = β_*j*+ 1_ *j* = *j*+ 1
Step 4: If *j*<*k*−1 back to Step 4, else continue
Step 5: Calculate the k smallest eigenvalue and eigenvector based on diagonal matrix *T*

### 2.4. Applying FCM algorithm to eigenvector matrix

The FCM algorithm (Nayak et al., 2014) allows a point to belong to one or more groups depending on the degree of membership function of each point corresponding to the centers of the groups (using fuzzy logic). Therefore, FCM has more flexibility with data sets with overlapping data clusters (high similarity with images). The algorithm is mainly based on the optimization of the objective function according to the formula (3).


(3) $$\begin{array}{l} J_{fcm}\left(Z,U,V\right)=\overset{C}{\underset{i=1}{\sum }}\overset{N}{\underset{j=1}{\sum }}u_{ij}^{m}||x_{j}-c_{i}|{|}^{2} \end{array}$$

In which, the matrix belonging to *U*=*[u*_*ij*_*]* ϵ *M*_*fcm*_ is the fuzzy partition of the data set Z, u_ij_ ϵ [0,1] indicates the dependence of point x_i_ on the j^th^ cluster, with $\overset{C}{\underset{j=1}{\sum }}u_{ij}=1,\forall i$; *V* = *[c*_1_*, c*_2_*, …, c*_*c*_*]* is the sample vector or cluster center of the C groups, calculated according to the distance standard${\;D}_{ij}=||x_{j}^{i}-c|{|}^{2}$; *m*∈[1;∞] is the exponent that determines the fuzziness of the clustering.

Instead of using k-mean in Ncut algorithm, we propose the FCM algorithm to group the image from second smallest eigenvalue by fuzzy original data to optimal cluster according to Algorithm 4. The FCM are fuzzy-graph structures for representing data in a fuzzy way. It accepted a computation noise for clustering, and it is more optimal on the real data, and eigenvector set from the Ncut problem.

**Algorithm 4:** FCM_Ncut.

**Input:**k eigenvector matrix
**Output:**Clustered k eigenvector matrix
Step 1: Let the matrix U ϵ R^nxk^ be the matrix
with k eigenvectors v_1_, …, v_k_ in column form
Step 2: For i = 1, …, n, let y_i_ ϵ R^k^ be
the vector corresponding to the i^th^ row of the matrix U
Step 3: Group the points (y_i_)_i = 1, …, *n*_ ϵ R^k^ using FCM
into clusters C_1_, …, C_k_
Step 4: We have the result as clusters A_1_, …,
A_k_ with A_i_ = {j/ y_j_ ϵ C_i_}

## 3. Experimental result

We setup the algorithm in the personal computer which had the Intel core i5 CPU (2.3 GHz), 8 GB RAM, GeForce GT 1050Ti, window 10 professional 64. We coded parallel in CUDA (CUDA Toolkit v9.0 and visual studio 10.0) and then implemented in MATLAB (R2016b). Our data (http://decsai.ugr.es/cvg/dbimagenes) included 15 images 128 × 128, 37 images 256 × 256, 64 images 512 × 512, and 3 images 1,024 × 1,024.

### 3.1. Determination of number of clusters in segmentation

We conduct an experiment of determination of number of clusters in segmentation on four datasets. The comparison is processed on the resultant number of clusters from intuition and our method on histogram. The Table 1 is the demonstration of our experiment on 4 datasets with the threshold of height deviation and peak distance of δ_1_ and δ_2_ as 50 and 100 respectively.

**Table 1:** The number of clusters from an image by intuition and K-segment_Histogram algorithm on using histogram.

**Data set**	**Number of correct images**	**Number of false images**	**Average number of different clusters**
1	11	4	1.5
2	31	6	1.3
3	57	7	1.5
4	3	0	0

From Table 1, it can be seen that the difference between the two ways of visual determination and that using histogram is not significant. Therefore, the prediction of cluster number is feasible in dynamic image processing applications.

### 3.2. Computing the similarity matrix W

**Figure 4:**
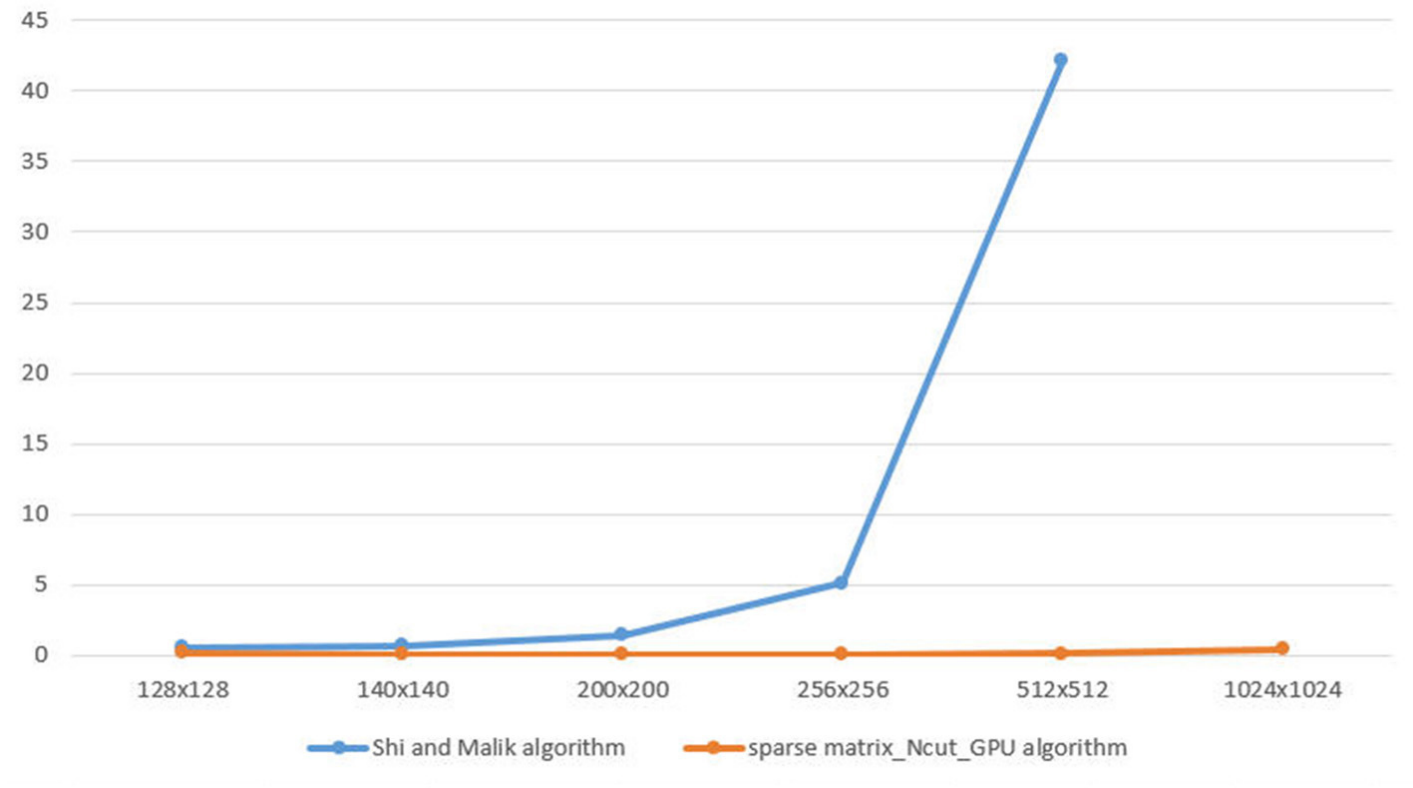
The speed time between Shi/Malik algorithm and sparse matrix_Ncut_GPU algorithm.

As Figure 4, we have the graph illustrates the speed time to calculate W matrix between Shi and Malik algorithm and sparse matrix_Ncut_GPU algorithm.

The vertical axis is the speed(s) and the horizontal axis is the size of image which is demonstrated in Table 2.

**Table 2:** The demonstration of speed time to calculate matrix W between Shi/Malik and sparse matrix_Ncut_GPU algorithm.

**Size**	**Runtime(s)**	
	**Shi/Malik**	**Sparse matrix_Ncut_GPU**
128 x 128	0.57	0.16
140 x 140	0.66	0.03
200 x 200	1.41	0.04
256 x 256	5.13	0.05
512 x 512	42.13	0.13
1,024 x 1,024	NA	0.43

In the graph, when image is 1024 × 1024, the Shi and Malik method is being out of memory. The sparse matrix_Ncut_GPU algorithm executes for a small increase when the image size increases.

### 3.3. Computing the matrix's eigenvalues

In this section, the experiment is conducted on the increasing image size in Table 3 on the three clusters (*k* = 3). This corresponds to exploring three eigenvalue and eigenvector of the W matrix. The Table 3 represents us that the GPU time (time to find eigenvalue in parallel way) is quicker than Shi/Malik method.

**Table 3:** The demonstration of speed time to calculate eigenvalues between Shi/Malik method and Lanczos_Ncut_GPU method.

**Size**	**Runtime(s)**	
	**Calculating eigenvalues in the Shi/Malik algorithm**	**Calculating eigenvalues in the Lanczos_Ncut_GPU algorithm**
128 x 128	1.63	0.69
140 x 140	2.36	0.85
200 x 200	6.21	0.94
256 x 256	17.34	0.97
512 x 512	NA	1.28
1,024 x 1,024	NA	2.84

**Figure 5:**
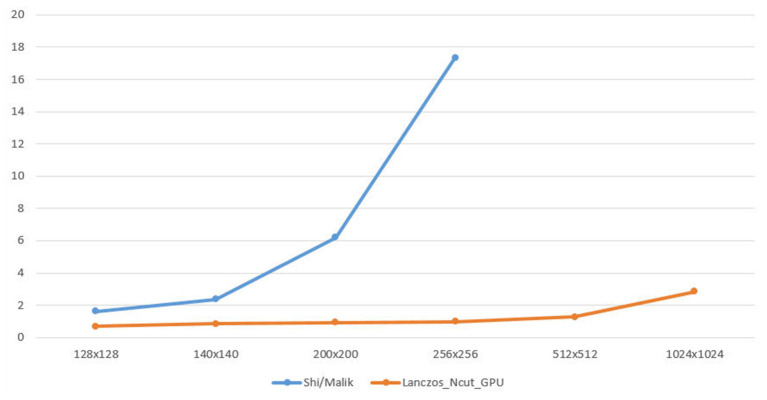
Calculating eigenvalues in the Shi/Malik method and Lanczos_Ncut_GPU method with size of image (3 segment).

The graph below illustrate the Shi/Malik method and Lanczos_Ncut_GPU method with size of image, the number of segment as shown in Figure 5.

The result of calculating eigenvalues by Lanczos_Ncut_GPU algorithm is 17 times faster than that by Shi/Malik algorithm when the image size increases.

### 3.4. Grouping on the eigenvalue by k–mean and FCM

**Figure 6:**
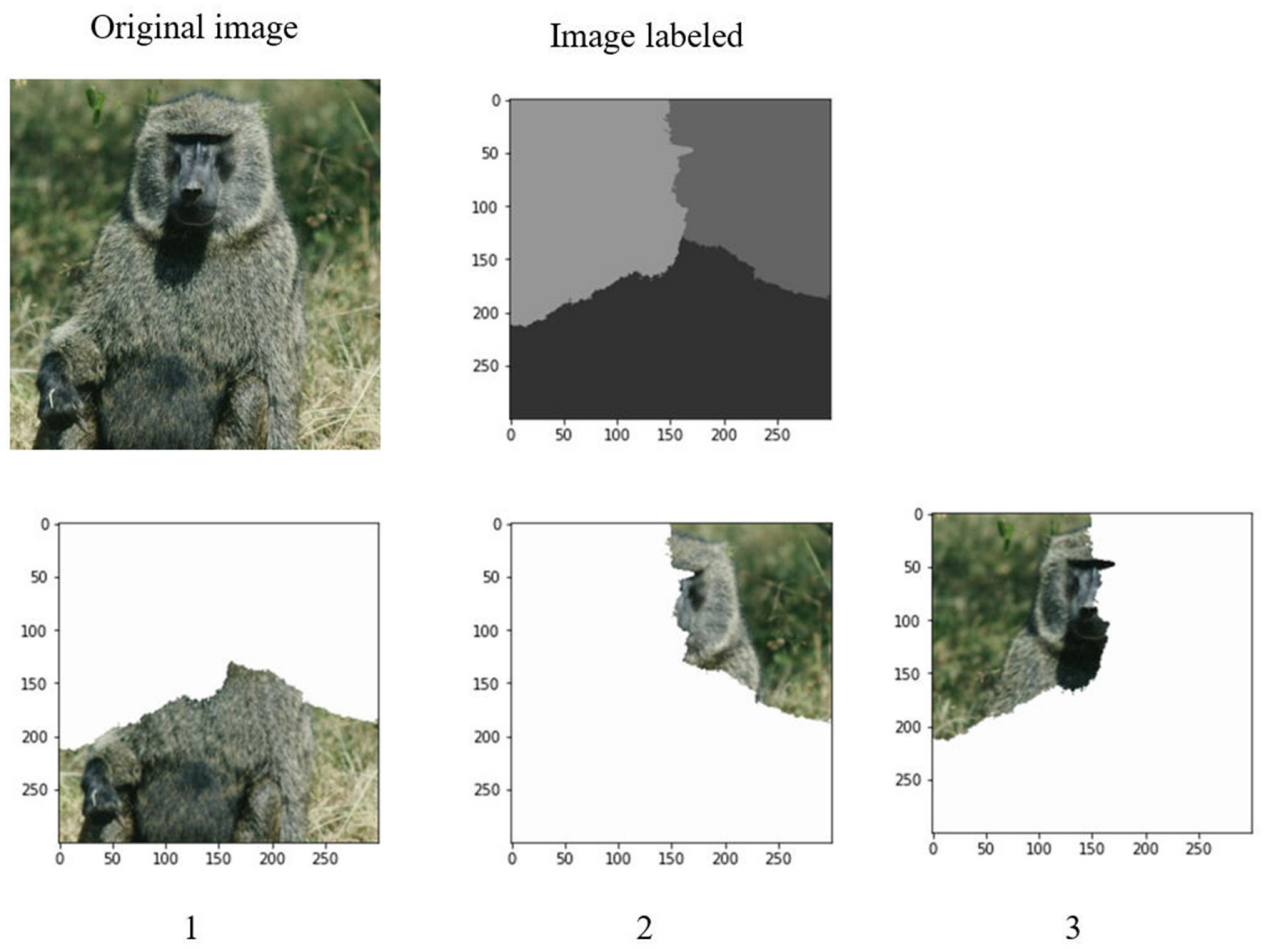
Grouping on the eigenvalue by k-mean to k classes.

**Figure 7:**
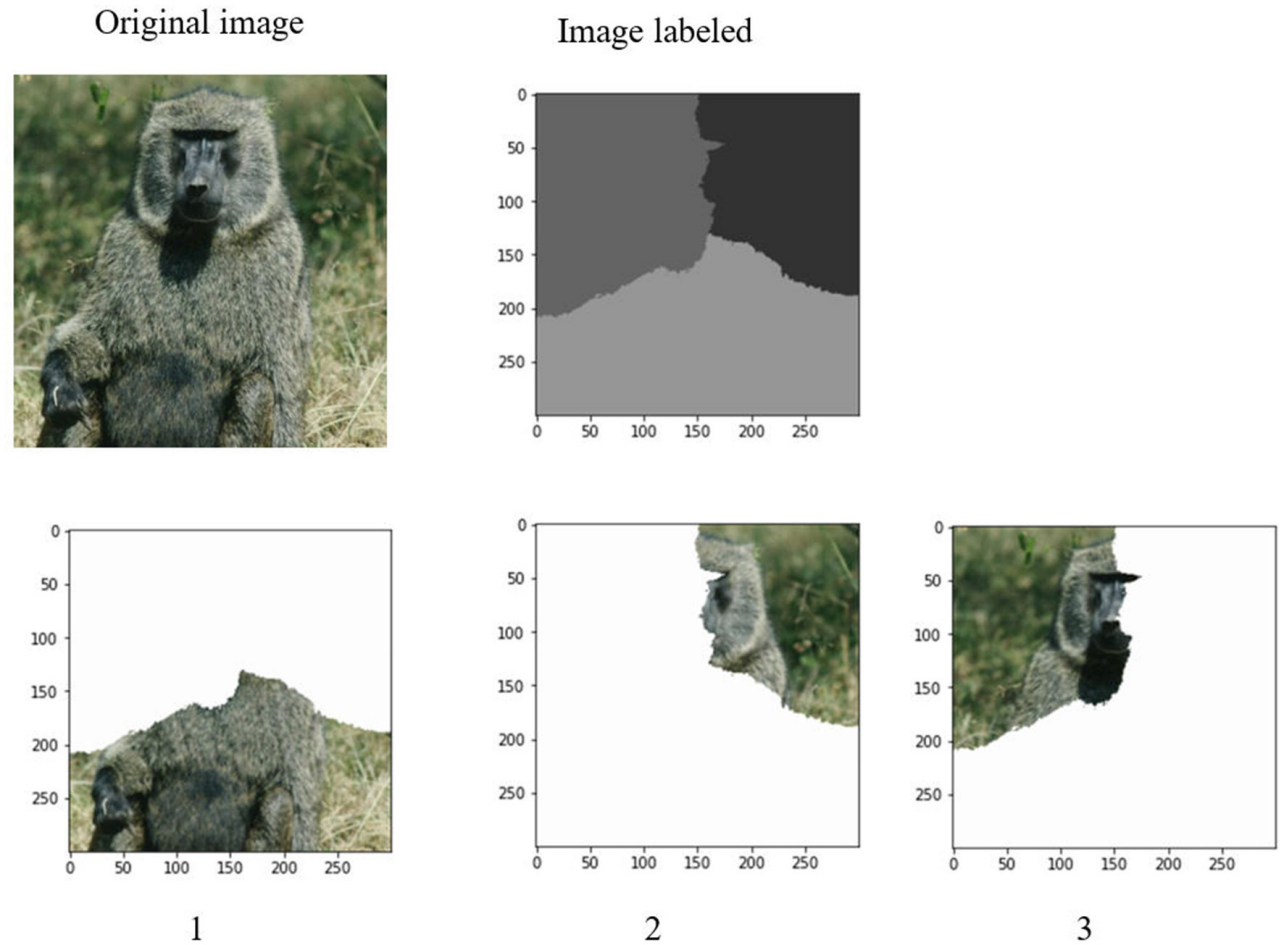
Grouping on the eigenvalue by FCM to k classes.

**Figure 8:**
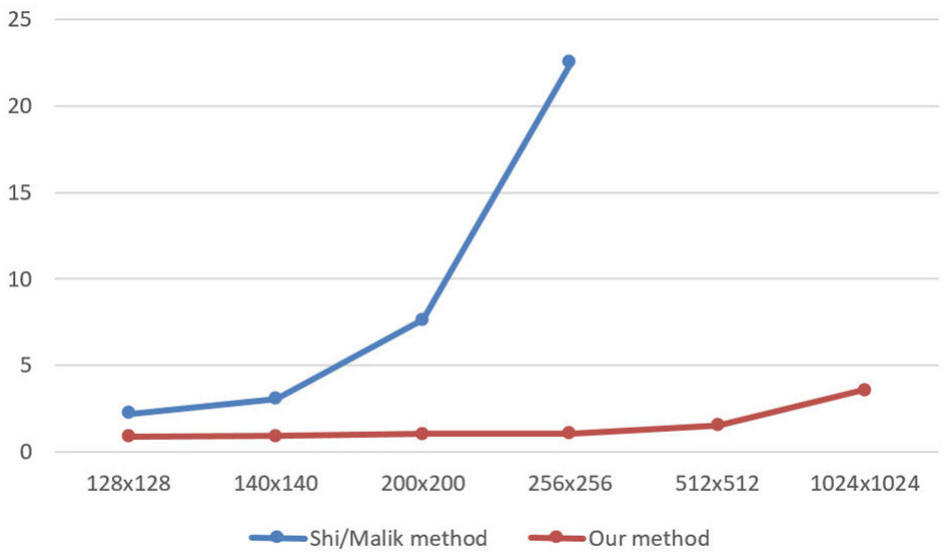
The speed time to calculate image segmentations between Shi/Malik algorithm and Our algorithm.

After determination of a set of eigenvalues, the Ncut algorithm makes use of these eigenvalues to cluster them into k clusters. It corresponds to the k regions of an image. Figures 6, 7 demonstrate the clustering results by K-means and FCM. There are no differences between the two approaches to image segmentation. Figure 8 and Table 4 depict the computational time in image segmentation between Shi/Malik's algorithm and the one we improved by GPU. It is found that the execution time of the algorithm we improved by GPU is 20 times faster than that of the Shi/Malik algorithm while keeping the same accuracy.

**Table 4:** The demonstration of speed time to calculate image segmentations between Shi/Malik method and our method by GPU.

**Size**	**Runtime(s)**	
	**Shi/Malik**	**Sparse matrix_Ncut_GPU**
128 x 128	2.22	0.88
140 x 140	3.04	0.92
200 x 200	7.67	1.04
256 x 256	22.51	1.09
512 x 512	NA	1.56
1,024 x 1,024	NA	3.58

## 4. Conclusion

In this paper, we analyze the Ncut algorithm to cluster an image into regions. Because in the Ncut algorithm, we have to input the number of clusters k, it is not automated process. For that reason, we predict k group depending on histogram will give a good result. Moreover, we improve the speed performance by parallel computing on GPU. Specifically, it is to parallelize the calculation of the W matrix and finding of the eigenvector matrix in the Ncut algorithm. Finally, we used the FCM algorithm to cluster the data in the above eigenvector matrix. Some experimental results on image data sets are conducted to prove that our approach is 20 times quicker than Shi and Malik approach in computing eigenvalue and computing similarity matrix.

## Data availability statement

Publicly available datasets were analyzed in this study. This data can be found here: https://ccia.ugr.es/cvg/dbimagenes.

## Author contributions

PB, HH, and NT have designed methods. NT implemented it and tested performance. All authors contributed to the article and approved the submitted version.

## References

[B1] Agulleiro J. I. Vázquez F. Garzón E. M. Fernández J. J. (2012). Hybrid computing: CPU+GPU co-processing and its application to tomographic reconstruction. Ultramicroscopy. 115, 109–114. 10.1016/j.ultramic.2012.02.003 22475372

[B2] Anderson R. J. Setubal J. (1992). “On the parallel implementation of goldbergs maximum flow aglrotithm,” in Proceedings of the 4th ACM Symp. on Parallelism in Algorithms and Architectures (SPAA) (Brazil), 168–177.

[B3] Augustine D. P. Raj P. (2016). Performance evaluation of parallel genetic algorithm for brain MRI segmentation in hadoop and spark. Indian J. Sci. Technol. 9, 1373. 10.17485/ijst/2016/v9i48/91373

[B4] Baker Q. B. Balhaf K. (2017). “Exploiting GPUs to accelerate white blood cells segmentation in microscopic blood images,” in Proceedings of the 8th International Conference on Information and Communication Systems (ICICS) (Irbid. Jordan), 136–140.

[B5] Belahcene M. Chouchane A. Benatia M. A. Halitim M. (2014). “3D and 2D face recognition based on image segmentation,” in Proceedings of the 2014 International Workshop on Computational Intelligence for Multimedia Understanding (IWCIM) (Paris, France).

[B6] Bell E. Garland M. (2008). Efficient sparse matrix-vector multiplication on CUDA. NVIDIA Technical Report NVR-2008–R-2004.

[B7] Cao J. Chen L. Wang M. Tian Y. (2018). Implementing a parallel image edge detection algorithm based on the otsu-canny operator on the hadoop platform. Comput. Intell. Neurosci. 20, 8284. 10.1155/2018/3598284 29861711 PMC5971336

[B8] Cullum J. K. Willoughby R. A. (2002). Lanczos Algorithms for Large Symmetric Eigenvalue Computations. SIAM: Society for Industrial and Applied Mathematics, Birkhäuser, Boston, United States.

[B9] Dalvand M. Fathi A. Kamran A. (2020). Flooding region growing: a new parallel image segmentation model based on membrane computing. J. Real-Time Image Process. 18, 37–55. 10.1007/s11554-020-00949-0

[B10] Dhanachandra N. Manglem K. Chanu Y. J. (2015). Image segmentation using k-means clustering algorithm and subtractive clustering algorithm. Procedia Comput. Sci. 54, 764–771. 10.1016/j.procs.2015.06.09027816803

[B11] Fakhi H. Bouattane O. Youssfi M. Hassan O. (2017). “New optimized GPU version of the k-means algorithm for large-sized image segmentation,” in Proceedings of the 2017 Intelligent Systems and Computer Vision (ISCV) (Fez, Morocc).

[B12] Li X. Song J. Zhang F. Ouyang X. Khan S. U. (2016). Mapreduce-based fast fuzzy C-means algorithm for large-scale underwater image segmentation. Fut. Gen. Comp. Sys. 65, 90–101. 10.1016/j.future.2016.03.004

[B13] Liu B. He S. He D. Zhang Y. Guizani M. (2019). A spark-based parallel fuzzy C-means segmentation algorithm for agricultural image big data. IEEE access. 7, 42169–42180. 10.1109/ACCESS.2019.2907573

[B14] Minaee S. Wang Y. (2019). An admm approach to masked signal decomposition using subspace representation. IEEE Transact. Image Process. 28, 3192–3204. 10.1109/TIP.2019.2894966 30703020

[B15] Nayak J. Naik B. Behera H. S. (2014). “Fuzzy C-Means (FCM) Clustering algorithm: a decade review from 2000 to 2014,” in Proceedings of the Computational Intelligence in Data Mining. Smart Innovation, Systems and Technologie (Springer, New Delhi), 2 133–149. 10.1007/978-81-322-2208-8_14

[B16] Nock R. Nielsen F. (2004). Statistical region merging. IEEE Trans. Pattern Anal. Mach. Intell. 26, 1452–1458. 10.1109/TPAMI.2004.110 15521493

[B17] Shi J. Malik J. (2000). Normalized cuts and image segmentation. IEEE Trans. Pattern Anal. Mach. Intell. 22, 888–905. 10.1109/34.868688

[B18] Shiloach Y. Vishkin U. (1982). An o(n^2^log n) parallel max-flow algorithm. J. Algor. 3, 128–146. 10.1016/0196-6774(82)90013-X

[B19] Sirotković J. Dujmić H. Papić V. (2012). “K-means image segmentation on massively parallel GPU architecture,” in Proceedings of the 35th International Convention MIPRO (Opatija. Croatia), 489–494.

[B20] Starck J.-L. Elad M. Donoho D. L. (2005). Image decomposition via the combination of sparse representations and a variational approach. IEEE Transactions on Image Processing. 14, 1570–1582. 10.1109/TIP.2005.852206 16238062

[B21] Wang N. Chen F. Yu B. Qin Y. (2020). Segmentation of large-scale remotely sensed images on a Spark platform: a strategy for handling massive image tiles with the MapReduce model. ISPRS J. Photogram. emote Sens. 162, 137–147. 10.1016/j.isprsjprs.2020.02.012

[B22] Wang X. Pan J. S. Chu S. C. (2020). A parallel multi-verse optimizer for application in multilevel image segmentation. IEEE Access. 8, 32018–32030. 10.1109/ACCESS.2020.2973411

[B23] Wassenberg J. Middelmann W. Sanders P. (2009). “An Efficient parallel Algorithm for Graph-based Image segmentaion,” in Proceedings of the 2009 Computer Analysis of Images and Patterns (Springer, Berlin. Heidelberg), 1003–1010.

[B24] XianLou H. ShuangYuan Y. (2013). “Image segmentation based on Normalized Cut and CUDA parallel implementation,” in Proceedings of the 5th IET International Conference on Wireless, Mobile and Multimedia Networks (ICWMMN 2013) (Beijing China) 209–214.

[B25] Yu T. Lim S. N. Patwardhan K. Krahnstoever N. (2009). “Monitoring, recognizing and discovering social networks,” in Proceedings of the 2009 IEEE Conference on Computer Vision and Pattern Recognition (Miami, FL), 1462–1469.

