# Supplementary material for: A proposed scenario to improve the Ncut algorithm in segmentation
Source: Front Big Data. 2023 Mar 3;6:1134946. doi: 10.3389/fdata.2023.1134946 (PMC10020342; doi:10.3389/fdata.2023.1134946)
Supplement: Supplementary file 1 [file Data_Sheet_1.docx]

# Appendix

a. Mask construction

For example, if $r=2.5$, we get a mask like figure 9. Logical values of 1 represent positions within the circle centered at (0,0) with radius $r$. Specifically, the coordinates of the neighborhoods of (0, 0) within the circle of radius $r$ are {(-1,2), (0, -2), (0, -1), (0, 0), (0 , 1), (0, 2), (1, -2), (1, -1), (1, 0), (1, 1), (1, 2), (2, -1), (2, 0), (2, 1)}. As a result, coordinates of the neighborhoods of (0,0) converted into two vectors x and y are respectively: (-1, 0, 0, 0, 0, 1, 1, 1, 1, 1, 2, ,2, 2) and (2, -2, -1, 0, 1, 2, -2, -1, -1, 0, 1, 2, -1, 0 , 1).


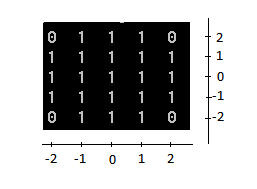


**Figure 9.** Example of a mask with a radius of 2.5

Figures 10a and 10b illustrate finding neighborhoods with any vertex i, we apply the created mask to i. Neighborhood coordinates are calculated by translating the coordinates of the neighborhoods of $(0,0)$ in terms of Ox and Oy a segment x and y where x and y are the coordinates of vertex i.


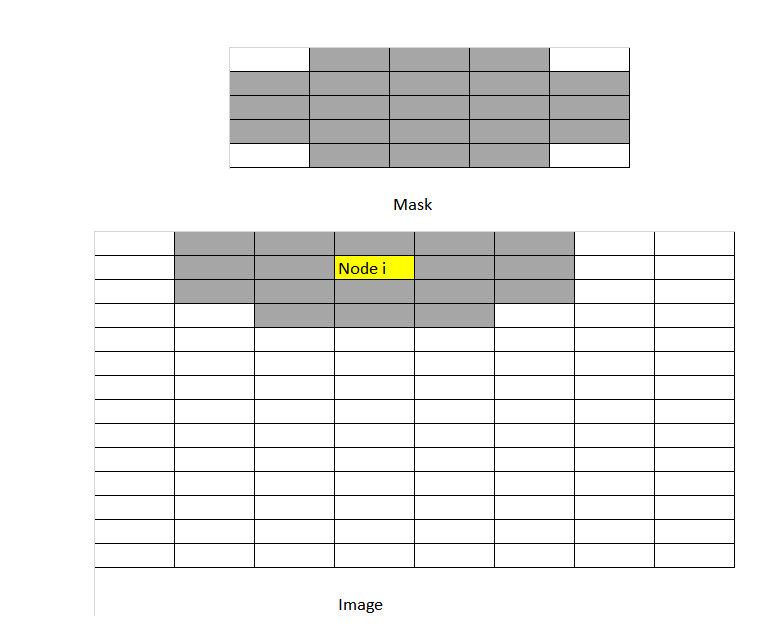


(a) Mask


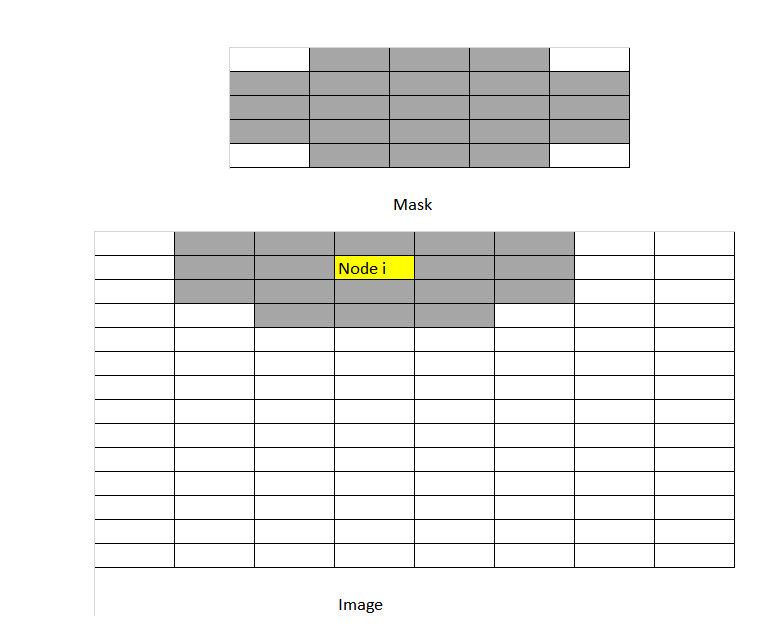


(b) All neighborhoods of vertex i

**Figure 10.** (a) Mask with a radius of 2.5; (b) All neighborhoods of vertex i

b. Algorithm to calculate the number of neighboring points with random vertex

We build algorithm 5 to calculate the number of vertices associated with a random vertex i without looping through the use of a mask.

**ALGORITHM 5**

**Input**: Radius r, given vertex coordinates (xpos,ypos), size of image (nrow, ncol)

**Output**: Total number of vertices associated with a given vertex

Begin

k = floor(r)

TL = (0 > (k - xpos))?0:(k-xpos)

if(xpos + k >= Row )

BL = xpos + k + 1 - Row

else

BL = 0

end if

LL = (0 > (k - ypos))?0:(k-ypos)

if(ypos + k >= Col)

RL = ypos + k + 1 - Col

else

RL = 0

end if

xtop = (k - TL)*(-1)

yleft = (k- LL)*(-1)

xbot = (k - BL)

yright = (k - RL)

numTL = lost(TL)

numBL = lost(BL)

numLL = lost(LL)

numRL = lost(RL)

temp = intersect(xtop,xbot,yleft,yright)

number = sum() – numTL – numBL – numLL – numRL + temp

End

c. Algorithm to calculate the number of non-zero elements of W

**ALGORITHM 6 (FindAmountNumber)**

**Input**: Size of the image ($row\times col$), radius r to build the mask

**Output**: Number of non-zero elements of W

Step 1: Initialize an Amount array of size $row\times col$ to store neighboring sets of each vertex

Step 2: Copy Amount array from CPU to GPU

Step 3: Initialize the number of flows used as $row\times col$

Step 4: Gọi hàm kernel trong GPU để thực thi song song các luồng

Step 4.1: Each flow in turn uses algorithm 5 to calculate the number of neighbors at each pixel, storing it in the Amount array

Step 4.2: The number of non-zero elements of W is the sum of the terms of the Amount array

d. Algorithm for multiplying two vectors in GPU

**ALGORITHM 7 (Multiply_vector_vector)**

**Input**: x,y are the two vectors that need to be multiplied in the GPU, respectively, and n is the number of elements of the vector

**Output**: Result of x * y

Step 1: Build a grid of execution threads (gridDim = (n, 1, 1), blockDim = (1, 1, 1))

Step 2: Allocate memory for the result variable

Step 3: Call the kernel function to calculate the result of multiplying vector and vector

Step 3.1: Determine the index of each thread

idx = blockidx.x*blockDim.x + threadIdx.x

Step 3.2: In each thread execute the multiplication:

result += x[idx] * y[idx]

result is the result to be calculated

e. Matrix multiplication algorithm with vector in GPU

**ALGORITHM 8 (Multiply_matrix_vector)**

**Input**: d and y are respectively matrix and vector to be multiplied in GPU respectively; n and m are respectively the number of elements of matrix and vector

**Output**: Result of d * y

Step 1: Build a grid of threads (gridDim = (n, 1, 1), blockDim = (1, 1, 1))

Step 2: Allocate memory for the result variable

Step 3: Call the kernel function to calculate the result of matrix-vector multiplication

Step 3.1: Determine the index of each thread

idx = blockidx.x*blockDim.x + threadIdx.x

Step 3.2: In each thread of multiplication:

if idx<n then

for (int k = 0; k < m; k++)

result[idx] += d[idx* m + k] * y[k]

‘result’ is the vector to be calculated

f. Algorithm for subtracting two vectors in GPU

**ALGORITHM 7 (Subtract_vector_vector)**

**Input**: x,y are the two vectors that need to be subtracted in the GPU, respectively, and n is the number of elements of the vector

**Output**: Result of x - y

Step 1: Build a grid of execution threads (gridDim = (n, 1, 1), blockDim = (1, 1, 1))

Step 2: Allocate memory for the result variable

Step 3: Call the kernel function to calculate the result of subtracting vector and vector

Step 3.1: Determine the index of each thread

idx = blockidx.x*blockDim.x + threadIdx.x

Step 3.2: In each thread execute the subtraction:

result[idx] = x[idx] - y[idx]

result is the result vector to be calculated
